# The First Case of Schaumann Bodies in Pediatric Very Early Onset Crohn’s Disease: Case Report and Literature Review

**DOI:** 10.3390/children11101216

**Published:** 2024-10-06

**Authors:** Jovan Jevtić, Miloš Đuknić, Nevena Popovac, Nina Ristić, Ivan Milovanovich, Milica Radusinović, Irena Đordjić, Ljubica Simić, Gorana Nikolić, Maja Životić, Ana Mioljević, Nikola Bogosavljević, Radmila Janković

**Affiliations:** 1 Institute of Pathology ‘Prof. Dr. Đorđe Joannović’, Faculty of Medicine, University of Belgrade, 11000 Belgrade, Serbia; jovan.jevtic@med.bg.ac.rs (J.J.); milos.djuknic@med.bg.ac.rs (M.Đ.); ljubica.simic@med.bg.ac.rs (L.S.); gorana.nikolic@med.bg.ac.rs (G.N.); maja.zivotic@med.bg.ac.rs (M.Ž.); anamioljevic@gmail.com (A.M.); 2 University Children’s Hospital Tiršova, Faculty of Medicine, University of Belgrade, 11000 Belgrade, Serbia; nevena.popovac@udk.bg.ac.rs (N.P.); nina.ristic@udk.bg.ac.rs (N.R.); ivan.milovanovic@udk.bg.ac.rs (I.M.); milica.radusinovic@udk.bg.ac.rs (M.R.); irena.djordjic@udk.bg.ac.rs (I.Đ.); 3 Institute for Orthopedic Surgery “Banjica”, Faculty of Medicine, University of Belgrade, 11000 Belgrade, Serbia; nikola.bogosavljevic@med.bg.ac.rs

**Keywords:** Crohn’s disease, Schaumann bodies, transmural inflammation, granulomas, pediatric patients, chronic diarrhea, anemia

## Abstract

Crohn’s disease (CD) is a chronic inflammatory bowel condition with increasing global incidence. Diagnosing CD is challenging and requires close collaboration between clinicians and pathologists due to the lack of specific diagnostic criteria. Histologically, CD is characterized by transmural inflammation, crypt distortion, metaplasia, and granulomas, although granulomas are not always present. Schaumann bodies (SB), initially described in sarcoidosis, are rare in CD but have been reported in about 10% of cases. This case report presents a 4-year-old female with chronic hemorrhagic diarrhea, severe anemia, and elevated inflammatory markers. Endoscopic and histological evaluations suggested CD, with the presence of SB in the gastric mucosa. Further investigations ruled out sarcoidosis, confirming a diagnosis of multi-segmental, very early onset CD with atypical histological features. SB are inclusions composed of calcium carbonate crystals and conchoid bodies, typically found within giant cells. The presence of SB in the mucosa is rare, limiting their diagnostic significance in endoscopic biopsies. Differential diagnosis should exclude other granulomatous diseases such as intestinal tuberculosis and sarcoidosis. This case highlights the importance of considering SB in the diagnosis of CD, particularly in pediatric patients.

## 1. Introduction

Crohn’s disease (CD) is a chronic inflammatory bowel condition that can affect any part of the gastrointestinal tract, with periods of remission and exacerbation. The incidence of CD is on a constant rise globally, with significant increases reported over the past few decades [1,2]. Diagnosing CD is highly challenging. Although there are numerous diagnostic criteria, none are pathognomonic, necessitating a close collaboration between clinicians and pathologists [3]. The histological appearance of CD is characterized by inflammation that discontinuously affects the mucosa as well as deeper layers of the bowel wall (transmural inflammation). There is crypt distortion, Paneth cell and pseudopyloric metaplasia, fissures, granulomas, and other features. Although not specific to CD, the presence of granulomas is one of its most important characteristics [4]. However, granulomas are not always present in biopsies from patients with CD, and their prevalence varies significantly with age. In pediatric cases, granulomas can be seen in 30–60% of biopsies, while in adult cases, they are present in 15–36% of biopsies [5].

Schaumann bodies (SB) were first described by Schaumann in 1917 in sarcoidosis, and their nature was first elucidated by Williams in 1960 [6]. In 1963, the same author reported the presence of SB in CD, noting that they can be seen in about 10% of patients with CD [7]. Although granulomas are often seen in biopsies from children with CD [8,9], SB have not yet been described in granulomas of children with very early onset CD.

## 2. Case Report

**Figure 1:**
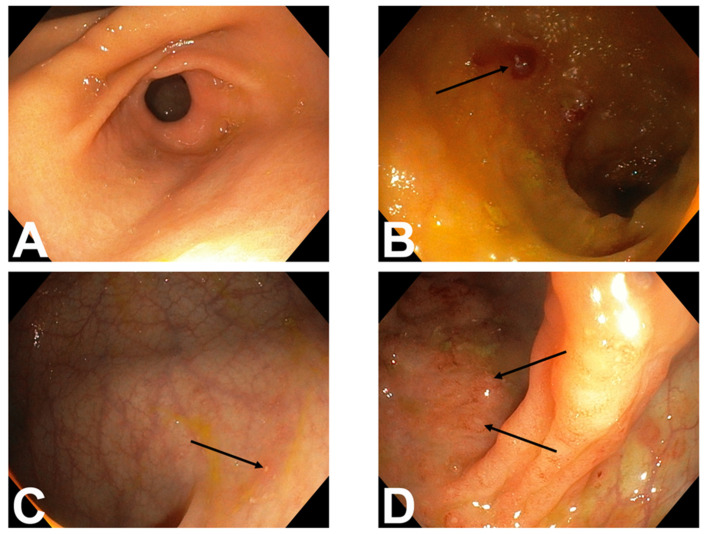
Endoscopic findings: (**A**)—Normal stomach; (**B**)—Ulceration in the terminal ileum (arrow); (**C**,**D**)—Numerous aphthae in the left and right halves of the colon (arrows).

We are presenting a case of our 4 years old female patient who presented with chronic haemorrhagic diarrhoea lasting over 6 months associated with severe sideropenia and anemia. By her pediatrician in primary health care she was repetitively prescribed with different antibiotics together with iron supplements, with partial therapeutic effect. Her physical examination revealed malnourished, pallor girl (BMI 15.3 kg/m^2^) with abdominal tenderness upon palpation. Her laboratory results showed persistent, iron refractory anemia (Hgb 109 g/L, MCV 70.2 fL) with hypoalbuminemia (30 g/L) and elevated serum inflammatory parameters (CRP 8 g/L, ESR 45 mm/h). Liver and kidney function tests were normal. Significant elevation of fecal calprotectin was present (1338 µg/g). Stool cultures excluded infectious etiology of intestinal symptoms. Her immunology panel revealed hypergammaglobulinemia (20.1 g/L). IgA was elevated (2.63 g/L), while IgM was normal (0.85 g/L). C3 (1.4 g/L) and C4 (0.36 g/L) levels were both normal. ANA was positive, showing ANA-HEp2 speckled in nucleoplasm (1:320) and dividing cells (1:320). Anti-dsDNA, AMA, ASMA, anti-LKM1, ANCA, SSA, and SSB were all negative. Screening for coeliac disease included serology (normal total IgA, negative TGA IgA) and duodenal biopsies (four from D2 and two from the bulb). The villi-to-crypt ratio was normal, the number of intraepithelial lymphocytes was within the normal range, and inflammatory cells in the lamina propria were within reference limits. As a result, coeliac disease was excluded. Bowel ultrasound showed thickening of ileal and colonic wall together with mesenterial lymphadenopathy and creeping fat. The patient underwent an esophagogastroduodenoscopy followed by a colonoscopy. Macroscopic findings of the upper endoscopy appeared normal. Endoscopic findings revealed terminal ileal ulcerations and focal aphthous signs of activity in the entire colon, overall highly suggestive of CD, with an anal fissure noted at the 6 o’clock (Figure 1).

**Figure 2:**
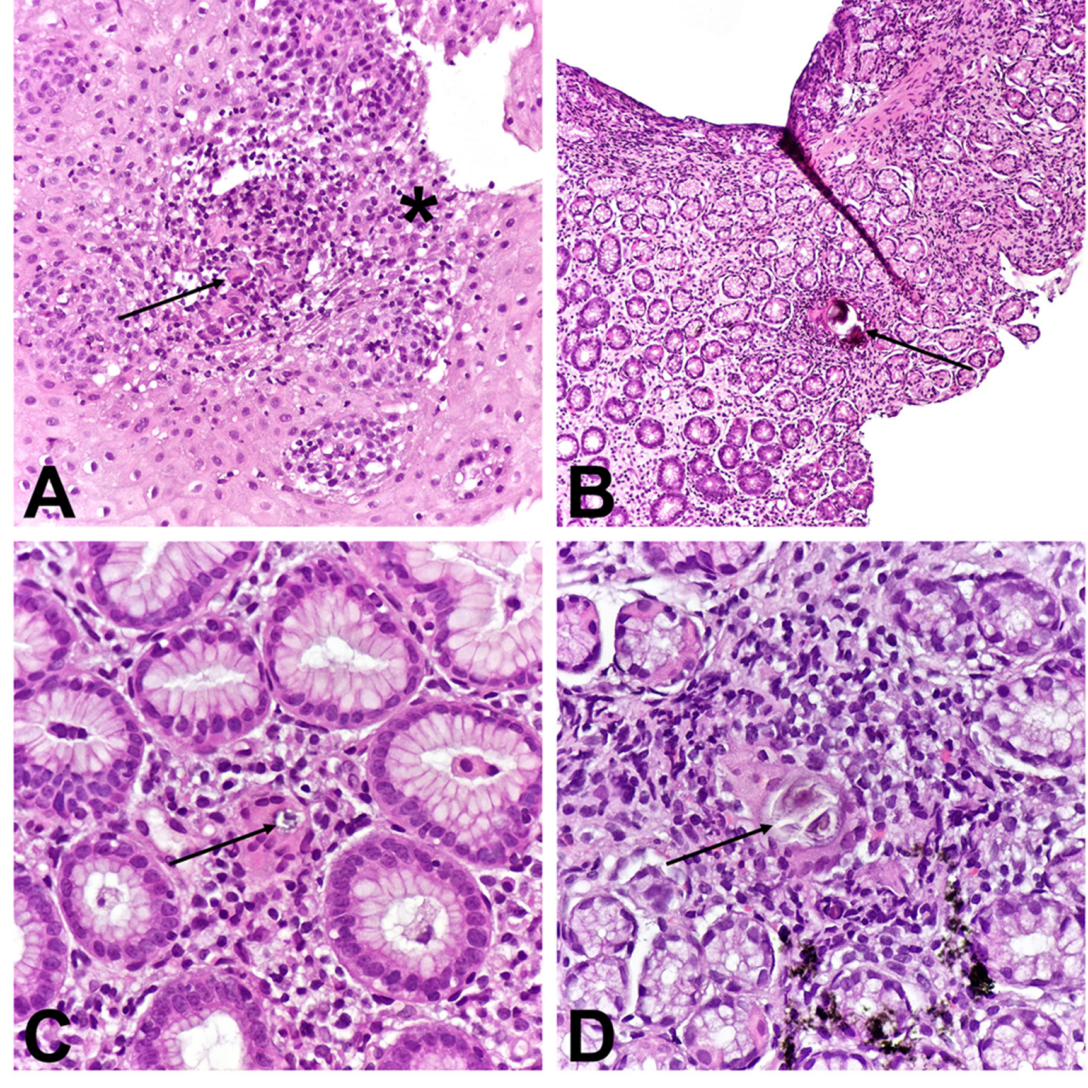
Histological findings of upper endoscopy: (**A**)—Macrophage aggregate (arrow), hyperplasia of the basal layer of the epithelium, dilation of intercellular spaces, and infiltration by numerous lymphocytes (asterisk); (**B**–**D**)—Granulomas with Langhans-type giant cells containing mixed-type SB in antrum (arrows).

**Figure 3:**
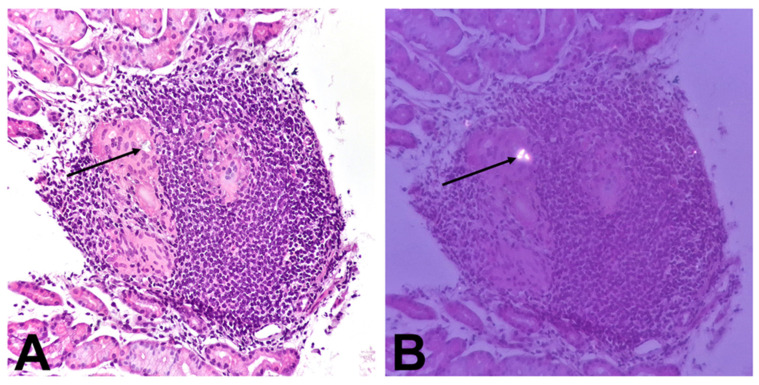
Histological finding in a biopsy of the corpus: (**A**)—Langhans-type giant cells containing mixed-type SB (arrow); (**B**)—SB within the cytoplasm of the giant cell exhibited birefringence after exposure to polarized light (arrow).

**Figure 4:**
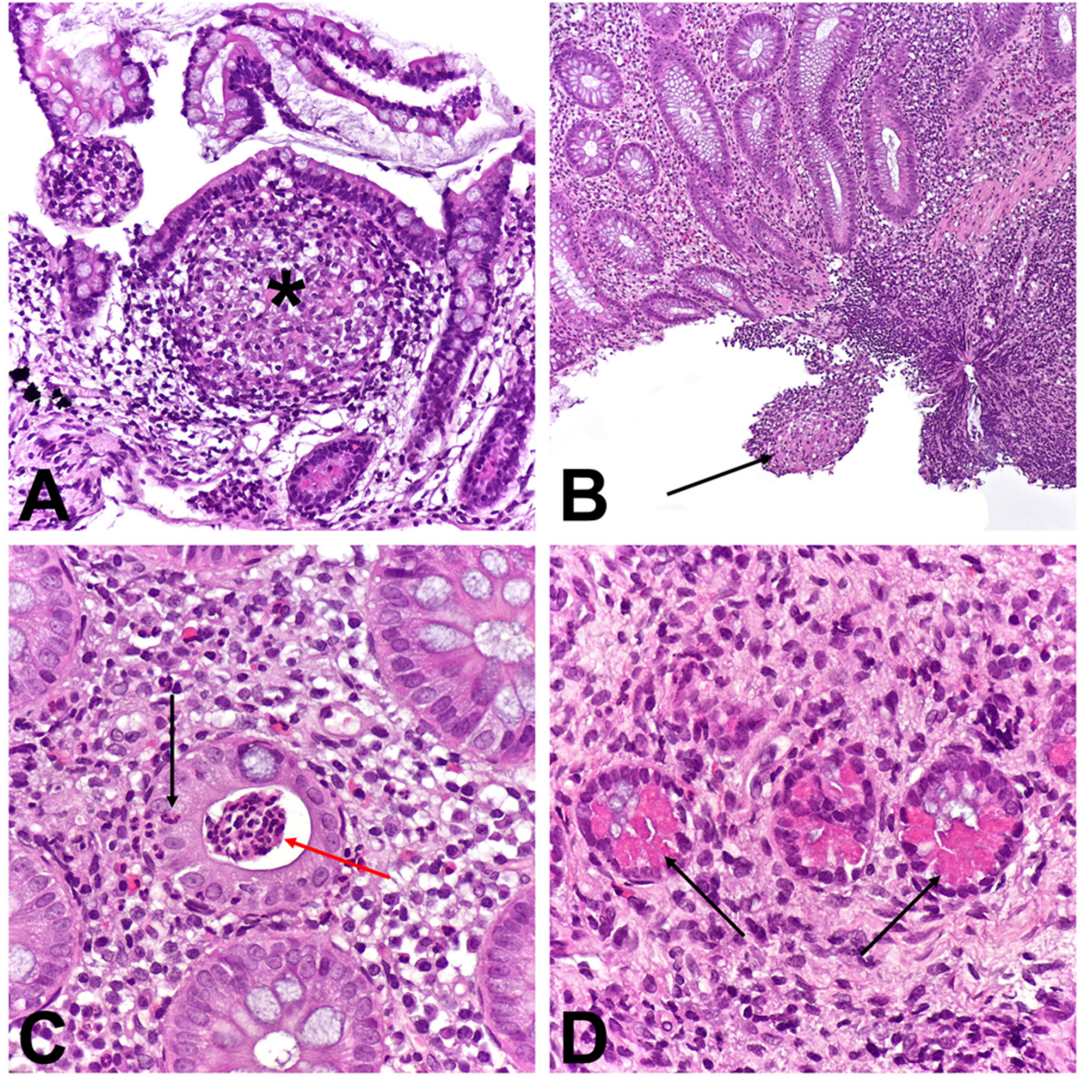
Histological findings of lower endoscopy: (**A**)—Granuloma in lamina propria of ileum (asterisk); (**B**)—Granuloma in submucosa of cecum (arrow); (**C**)—Cryptitis (black arrow) and crypt abscess (red arrow); (**D**)—Paneth cell metaplasia in distal parts of colon (arrow).

Biopsies were taken from all endoscopically examined parts of the GI tract. Histological analysis showed hyperplasia of the basal layer of the esophageal epithelium, dilated intercellular spaces, and infiltration by a greater number of lymphocytes. Additionally, a macrophage aggregate localized basally was observed (Figure 2A). Biopsies of the stomach revealed focal active pangastritis as well as multiple granulomas with Langhans-type giant cells containing mixed-type Schaumann bodies were found in the lamina propria of the mucosa. (Figure 2B–D). A SB within the cytoplasm of the giant cell localized in the lamina propria of the corpus exhibited birefringence after exposure to polarized light (Figure 3A,B). Duodenal biopsies showed normal characteristics. Histological analysis of lower endoscopy biopsies showed signs of Crohn’s ileocolitis with numerous erosions and ulcerations. Numerous granulomas without SB were observed in the lamina propria of the ileum (Figure 4A) and cecum (Figure 4B) as well as in descending colon. The crypts exhibited moderate distortion, numerous cryptitis, and crypt abscesses. (Figure 4C). In samples from the distal parts of the colon, Paneth cell metaplasia was also observed (Figure 4D). Lamina propria of the ileum and colon showed a dense inflammatory infiltrate composed of lymphocytes, plasma cells, eosinophils, and neutrophils, which was unevenly distributed.

Screening for tuberculosis with QuanTIFERON TB GOLD was negative. We also performed a sputum culture to further exclude tuberculosis. With both results being negative, tuberculosis was ruled out. Since SB are often seen in patients with sarcoidosis, further investigations were performed to exclude it. Angiotensin converting enzyme (ACE) was 67.7 U/L (normal range 29–112 U/L), urinary calcium was 0.8 mmol/L (normal range 0.15–7 mmol/L), and chitotriosidase was 60 nmol/mL/h (normal < 150 nmol/h/mL). To rule out pulmonary involvement that could be asymptomatic, an X-ray and a thoraco-abdominal CT were performed, but neither revealed any pathological changes. No skin lesions or lymphadenopathy suggestive of sarcoidosis were found. Ophthalmological examination was completely normal. Since no clinical and laboratory correlation for sarcoidosis was found, definitive diagnosis of multi-segmental, extensive, very early onset CD was confirmed with atypical and rare histological landmark. Exclusive enteral nutrition was used as initial induction of remission together with azathioprine (2.5 mg/kg/day) sequentially with biologics, Infliximab, which is about to be commenced. From the anamnesis, it was noted that the mother has a perianal fistula currently under investigation.

## 3. Discussion

SB are inclusion bodies seen in granulomatous diseases and are composed of calcium carbonate crystals and conchoid bodies (CB). Calcium carbonate exhibits birefringence under polarized light, while CB are characterized by their lamellation and basophilia. The aforementioned two structures can be observed in different proportions, thus SB can be classified into three types: crystalline SB, conchoidal SB, and mixed SB [10]. It is believed that calcium carbonate crystals serve as the primary nidus for the deposition of conchoidal bodies, which are composed of a protein matrix, calcium, phosphate, and iron [11]. SB are most commonly seen within epitheloid or Langhans-type giant cells, often occupying a large portion of the cell’s volume, but they can also be found intercellularly, typically as a result of cell rupture. The first description of SB was made by Swedish dermatologist Jörgen Nielsen Schaumann in a case of sarcoidosis in 1917 [12]. A more detailed description of SB was provided by Williams, who noted that SB are seen in sarcoidosis in up to 88% of cases, in berylliosis in 62% of cases, while in tuberculosis, SB can be seen in 6% of cases [6]. The same author first described the presence of SB in CD in 1964, noting that SB are seen in 10% of cases [7]. However, more recent studies indicate that the occurrence of SB is much more frequent and can be observed in nearly one-third of patients with CD. When it comes to distribution, SB are most commonly observed within the terminal ileum, seen in as many as 89.5% of cases, while in the colon they are seen in 7.9% of cases. Unfortunately, there are no data in the literature regarding their distribution within upper endoscopy biopsies. In terms of distribution across the layers of the intestinal wall, SB can be found in all layers but are most frequently observed within the muscular layer (92.1%), primarily between the circular and longitudinal muscle layers, followed by the subserosa (23.7%). SB are very rarely seen within the mucosa (2.6%), thus their diagnostic significance in endoscopic biopsies is very limited. Interestingly, in our case, Schaumann bodies (SBs) were exclusively localized within the mucosa of the stomach. According to the study by Liu et al., no difference was observed in the clinical characteristics between patients with Crohn’s disease (CD) with and without SBs. Additionally, the number of female patients with CD and Schaumann bodies was higher than that of males, which aligns with our case as our patient is female [10].

When it comes to differential diagnosis, CD with SB should be differentiated from other granulomatous inflammations, primarily intestinal tuberculosis (ITB) and sarcoidosis, as well as schistosomiasis.

Differentiating CD from ITB can be challenging due to overlapping clinical and histopathological features. Granulomas in CD are typically small, scattered, non-caseating and ill-defined. In contrast, granulomas in ITB are larger, confluent, and caseating, often densely packed and involving extensive areas of the intestinal wall. The presence of acid-fast bacilli within granulomas is a definitive marker for ITB, though not always detected in every biopsy, and can be confirmed through microbiological studies, including culture for Mycobacterium tuberculosis and PCR-based assays for detecting mycobacterial DNA [10]. Differentiating these two conditions is crucial because their treatment involves completely different forms of therapy, and incorrect diagnosis can lead to fatal outcomes.

To differentiate CD from sarcoidosis, particularly in the presence of SB, several key points must be considered. Granulomas are present in both conditions, but in sarcoidosis, they are typically intramucosal and non-caseating, whereas in CD, they are usually submucosal and may be associated with fistulas. Sarcoidosis lacks the mucosal architectural distortion and acute inflammation that is often seen in CD. Additionally, sarcoidosis often involves other organs with non-caseating granulomas [13,14].

Additionally, calcified Schistosoma eggs can closely resemble SB, especially CB, although their number is often much higher compared to SB. Furthermore, the eggs are most commonly observed in the mucosa and submucosa, have an oval shape, and the outer membrane can often be seen in the early stages of calcification [15].

## 4. Conclusions

Diagnosing CD is complex and requires close collaboration between clinicians and pathologists, particularly due to the presence of non-specific diagnostic criteria. SB, although commonly associated with sarcoidosis, can also be present in CD, including in very early onset cases. While usually seen within the muscular layer of the bowel, this case uniquely identified SB within the mucosa of the stomach antrum in case of a child. Our case highlights the importance of recognizing SB in CD to avoid misdiagnosis, especially with sarcoidosis.

## Data Availability

The original contributions presented in this report are included in the article. Further enquiries can be directed to the corresponding author.
